# Paper-Based Electrochemical Detection of Chlorate

**DOI:** 10.3390/s18020328

**Published:** 2018-01-24

**Authors:** Lisa C. Shriver-Lake, Dan Zabetakis, Walter J. Dressick, David A. Stenger, Scott A. Trammell

**Affiliations:** U.S. Naval Research Laboratory, Center for Bio/Molecular Science & Engineering (Code 6900), 4555 Overlook Avenue SW, Washington, DC 20375, USA; lisa.shriverlake@nrl.navy.mil (L.C.S.-L.); daniel.zabetakis@nrl.navy.mil (D.Z.); walter.dressick@verizon.net (W.J.D.); david.stenger@nrl.navy.mil (D.A.S.)

**Keywords:** chlorate, electrochemical detection, paper-based probe, electrocatalysis, polyoxometalates

## Abstract

We describe the use of a paper-based probe impregnated with a vanadium-containing polyoxometalate anion, [PMo_11_VO_40_]^5−^, on screen-printed carbon electrodes for the electrochemical determination of chlorate. Cyclic voltammetry (CV) and chronocoulometry were used to characterize the ClO_3_^−^ response in a pH = 2.5 solution of 100 mM sodium acetate. A linear CV current response was observed between 0.156 and 1.25 mg/mL with a detection limit of 0.083 mg/mL (S/N > 3). This performance was reproducible using [PMo_11_VO_40_]^5−^-impregnated filter paper stored under ambient conditions for as long as 8 months prior to use. At high concentration of chlorate, an additional catalytic cathodic peak was seen in the reverse scan of the CVs, which was digitally simulated using a simple model. For chronocoulometry, the charge measured after 5 min gave a linear response from 0.625 to 2.5 mg/mL with a detection limit of 0.31 mg/mL (S/N > 3). In addition, the slope of charge vs. time also gave a linear response. In this case the linear range was from 0.312 to 2.5 mg/mL with a detection limit of 0.15 mg/mL (S/N > 3). Simple assays were conducted using three types of soil, and recovery measurements reported.

## 1. Introduction

We are developing inexpensive, miniaturized, low-power electrochemical sensors for incorporation into autonomous unmanned aerial vehicles (UAVs), with the goal to identify explosives as well as their precursors and degradation products in the field [1,2,3]. Electroanalytical chemistry is a promising field in which instrumentation and assays can be further developed for the detection of explosives associated with improvised explosive devices (IEDs) and their manufacturing facilities. As current detection methods for nitrogen-based explosives improve, in terms of sensitivity and selectivity [4,5,6,7,8], focus is shifting to alternative explosives that are based on chlorates or peroxides [9].

Spectrophotometric methods for chlorate detection are currently amenable for field use. These methods are generally based on bleaching the color of a dye species [10,11,12,13,14] in the presence of chlorate and form the concept of commercial test kits. The color change may be visible to the naked eye or with the aid of very simple instrumentation, permitting development of a lightweight system that requires little or no power to operate. However, there remain specificity/selectivity issues since some of these colorimetric tests can respond to other oxidants.

Recent advances in electronics miniaturization and design can now provide lightweight, low-cost, rugged, low-power potentiostats [3,15], allowing electrochemical methods that exploit the redox behavior of the chlorate species to provide a more convenient alternative to spectrophotometric detection methods. Stopped-flow injection methods using amperometric or spectrometric techniques have been reported for the detection of chlorate in soil samples [16,17], and the catalytic electroreduction of chlorate by polyoxometalates has been demonstrated [18].

Polyoxometalates (POMs) are polyatomic anions formed from transition metal oxyanions linked together by their oxygen atoms into three-dimensional structures. The transition metal atoms are in their high oxidation states and can include Mo, W, V, Nb, or Ta. POMs have been studied as electrocatalysts [19,20], and in the case of polyoxotungstates and polyoxomolybdates the substitution of Mo or W by V increases the structure’s stability at higher pH and allows for the “tuning” of their electrochemical properties [19,21,22,23]. POMs are catalytic for the reduction of chlorate, and we have recently described the use and characterization of a multilayer film, prepared via layer-by-layer deposition of cationic para-rosaniline acetate dye and the vanadium-containing polyoxometalate anion, [PMo_11_VO_40_]^5−^, on indium tin oxide (ITO) as an electrode for determination of chlorate [1].

To miniaturize and modify the probe for incorporation into UAVs, we have taken advantage of paper-based electroanalytical chemistry combined with commercial screen-printed electrodes to construct simple probes for use in the field. The commercial screen-printed electrodes contain all three electrodes, i.e., the working, counter, and reference electrodes, needed for the major electroanalytical techniques, and are inexpensive, small, and robust enough for field work. The addition of the filter paper provides the volume for the electrolyte, making a practical thin layer cell for electrochemical detection. The paper can also serve as a filter for soil sampling or as a wipe for trace analysis. Here, the construction and demonstration of a simple paper-based/screen-printed electrode probe for the detection of chlorate in a variety of soil types is reported.

## 2. Materials and Methods

### 2.1. Materials

Ultrapure water of 18.2 MΩ·cm^−1^ resistivity obtained from a Milli-Q Advantage deionized water system was used to prepare all solutions and for all experiments. All chemicals were used as received. Sodium acetate, potassium chlorate, sodium chloride, potassium nitrate, sodium nitrite and sodium nitrate were all from Sigma-Aldrich Chemicals. Na_4_H[PMo_11_]VO_40_] was prepared with some modification of an earlier literature method [24] at a reduced scale recently reported in Trammell et al. [1]. Certified soil types where purchased from Sigma-Aldrich Chemicals.

### 2.2. Measurements

All electrochemical measurements were made under ambient, aerobic conditions. The cyclic voltammetry (CV), chronoamperometry, and chronocoulometry measurements were performed using a three-electrode configuration with a model 760 electrochemical workstation from CH Instruments. Screen-printed electrodes containing a carbon working electrode with a diameter of 2 mm, a carbon counter electrode, and a Ag/AgCl reference electrode were purchased from Pine Research Instrumentation. Digital simulations of the CVs were made with the CH Instruments software using a diffusive model with diffusion coefficients at 1 × 10^−5^ cm^2^/s and Butler–Volmer kinetics with alpha set to 0.5.

### 2.3. Paper-Based Electrochemical Probe

A solution of 1 mg/mL [PMo_11_VO_40_]^5−^ was prepared in a buffer containing 100 mM sodium acetate adjusted to pH 2.5 with concentrated HCl. An aliquot of the [PMo_11_VO_40_]^5−^ solution (50 µL) was added to a strip of Whatman #4 filter paper cut to dimension of approximately 1 cm × 1 cm. The paper was allowed to air dry and stored dry at room temperature until further use. For the electrochemical measurements, a single piece of filter paper containing [PMo_11_VO_40_]^5−^ was placed on top of a screen-printed electrode and 50 µL of sodium acetate (100 mM, pH 2.5) solution was added to the paper. For the chlorate dose–response, an additional 50 μL of potassium chlorate stock solution in sodium acetate (100 mM, pH 2.5) was added to the paper on top of the probe after 3 complete potential sweeps (0 to −1.0 V vs. Ag/AgCl and reversed) to equilibrate the paper-based probe and obtain a background signal. A total of 15 complete sweeps at 100 mV/s were run for each test sample, by which time the current had stabilized; the current was then read and recorded.

### 2.4. Soil Assay

To 100 mg of each soil type (clay, sand, loam soil), 100 µL of 2.5 mg/mL potassium chlorate in 100 mM sodium acetate pH 2.5 were added, and this mixture was placed under a stream of nitrogen until dry. For the assay, filter paper impregnated with the [PMo_11_VO_40_]^5−^ as above was placed on the electrode and wetted with 90 µL of acetate buffer. After 3 complete sweeps to equilibrate the paper-based probe and obtain a background signal, the dried soil was placed on top of the filter paper and another 90 µL of acetate buffer were added to fully saturate the soil samples. A total of 15 complete sweeps were run for each test sample to reach a stabilized current, after which the current was recorded as the response.

## 3. Results and Discussion

### 3.1. Detection of Chlorate

**Scheme 1:**
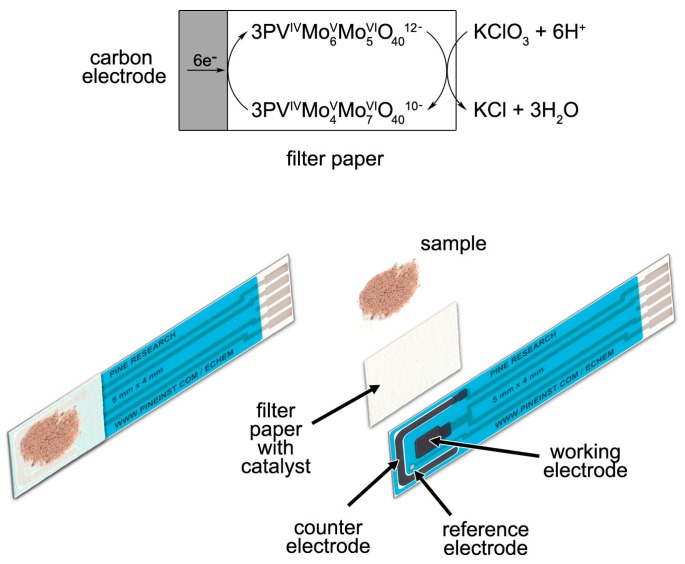
Paper-based electrochemical detection of chlorate.

The construction of a paper-based electrochemical probe containing the [PMo_11_VO_40_]^5−^ catalyst is simple and convenient when combined with screen-printed electrodes. A schematic is shown in Scheme 1. The filter paper containing the catalyst was cut into small strips that cover the electrode surface of the commercial screen-printed electrodes, and, with the addition of a small amount of buffer, the paper-based/screen-printed electrode became electrochemically active.

**Figure 1:**
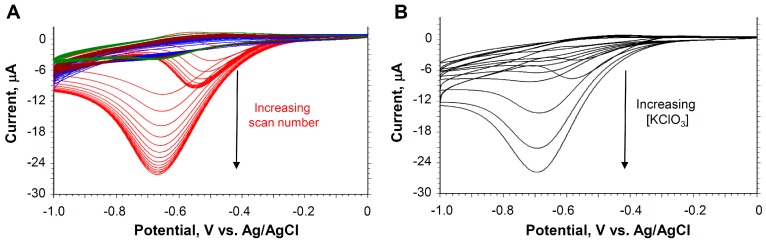
(**A**) Cyclic voltammograms with increasing scan number at 100 mV/s for the paper-based electrochemical probe containing 0.05 mg [PMo_11_VO_40_]^5−^ in 100 μL of buffer. Background (blue), NaNO_3_ (brown), KNO_3_ (green), KClO_3_ (red) all at 5 mg/mL. (**B**) Cyclic voltammograms with increasing [KClO_3_]from 0.078 to 5 mg/mL.

To characterize the electrochemical response of our paper-based probe, we used CV and chronocoulometry. As shown in Figure 1A, the CV of the paper-based/screen-printed electrode revealed a single redox peak at E_pc_ = −0.70 V vs. Ag/AgCl assigned to the reduction of the [PMo_11_VO_40_]^5−^ in the filter paper. The peak is broad and on a sloping background. The response is notably different from our earlier report of the layer-by layer system in which several well defined peaks are seen in the CVs [1]. In the earlier study, the electrode was directly modified with a film containing the catalyst. In the paper-based approach, the [PMo_11_VO_40_]^5−^ catalyst needed to diffuse to the electrode through the paper. The redox couple grows in after the addition of buffer and increases with scan number reaching a stable signal after 5 min.

The addition of KNO_3_ or NaNO_3_ had little effect on the CVs as shown in Figure 1A; however, with the addition of KClO_3_, there was a pronounced catalytic response at the cathodic peak. This peak grew as scan number increased, reaching a stable signal at 15 scans. The strong catalytic wave corresponds to the pH-dependent catalyzed reduction of chlorate to chloride, as shown in Scheme 1. Two one-electron reductions of Mo^VI^ components of the [PMo_11_VO_40_]^5−^ species generate the Mo^V^ oxidation state product, which is capable of reducing chlorate to chloride under acid conditions. The oxidized PV^IV^Mo_4_^V^Mo_7_^VI^O_40_^10−^ species produced is identical to the species serving as reactant. At the applied electrode potential, it is immediately reduced once again to PV^IV^Mo_6_^V^Mo_5_^VI^O_40_^12−^ and reacts with additional chlorate to continue the catalytic cycle.

**Figure 2:**
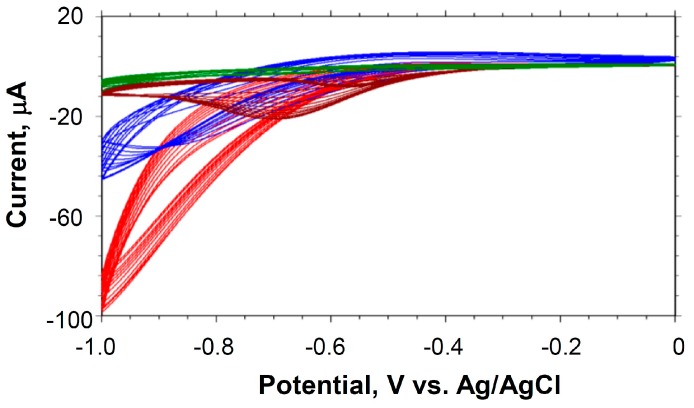
Cyclic voltammograms for the paper-based electrochemical probe in 100 μL of buffer containing 0.05 mg (green) of [PMo_11_VO_40_]^5−^ and with 5 mg/mL of KClO_3_ (brown) or 0.4 mg of [PMo_11_VO_40_]^5−^ (blue) and with 5 mg/mL of KClO_3_ (red).

Increasing the [PMo_11_VO_40_]^5−^ catalyst in the paper decreased the time needed to reach a stable signal and increased the catalytic current. However, the cyclic voltammograms became distorted with the higher catalyst concentration, shifting the cathodic peak to potentials of more negative values with the KClO_3_ signal appearing beyond the switching potential as shown in Figure 2.

**Figure 3:**
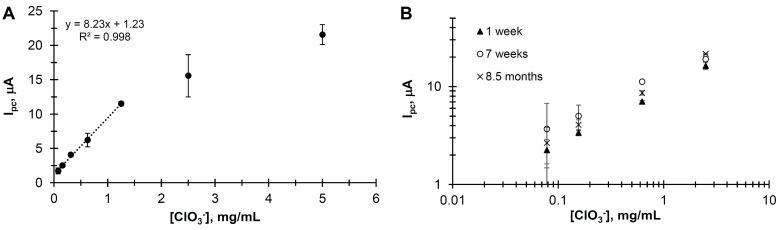
(**A**) Dose–response curve, I_pc_ vs. [KClO_3_]. Parameters: Scan rate = 100 mV/s; buffer = 100 μL of 50 mM sodium acetate adjusted to pH 2.5 with HCl; filter paper = 1 cm^2^ with 0.45 mg of [PMo_11_VO_40_]^5−^; electrodes = screen-printed carbon as working and counter with an Ag/AgCl screen-printed reference. (**B**) Cyclic voltammetry (CV) dose–response of the paper-based electrochemical probe as a function of the age of the filter paper containing the catalyst.

The catalytic response increased with increasing KClO_3_, as shown in Figure 1B. A plot of the dose–response curve, I_pc_ vs. [KClO_3_] is shown in Figure 3A, giving a linear range from 0.156 to 1.25 mg/mL with a detection limit of 0.083 mg/mL (S/N > 3) (Table 1).

Similar results within experimental error were observed for the [PMo_11_VO_40_]^5−^-impregnated filter paper stored under ambient conditions for as long as 8 months (Figure 3B) prior to use, further attesting to the stability and reproducibility of our system.

**Figure 4:**
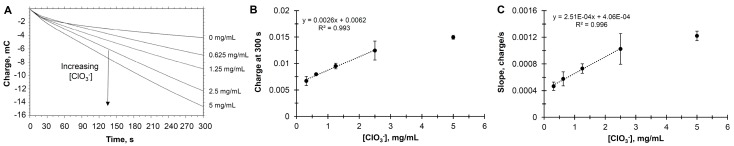
(**A**) Chronocoulometry with increasing [KClO_3_] for the paper-based electrochemical probe containing [PMo_11_VO_40_]^5−^ measured at −1 V vs. Ag/AgCl. (**B**) Dose–response curve, mC vs. [KClO_3_] (**C**) Dose–response curve, slope (mC/s) vs. [KClO_3_].

To investigate whether other techniques could improve the analytical response, chronocoulometry (Figure 4A) was investigated using total charge passed during 5 min or the slope (charge/s). A plot of the total charge passed at 5 min vs. [KClO_3_] is shown in Figure 4B, giving a linear range from 0.625 to 2.5 mg/mL with a detection limit of 0.31 mg/mL (S/N > 3). A plot of the final slope (mC/s) vs. [KClO_3_] is also shown in Figure 4C with a linear range from 0.312 to 2.5 mg/mL and a detection limit of 0.15 mg/mL (S/N > 3). Parameters for the different electroanalytical techniques are given in Table 1. Among the three cases, CV has the lowest LOD.

With an LOD of 0.083 mg/mL, our paper based-probe is less sensitive compared to our earlier work using CV for chlorate detection with a multilayer film containing [PMo_11_VO_40_]^5−^ on indium tin oxide (LOD = 0.027 mg/mL) [1]. However, the paper-based probe provides a more tractable construction of the electrode probe containing the catalyst. Both electrochemical techniques are less sensitive compared to elaborate instrumentation using amperometric (LOD = 0.00014 mg/mL) [16] or spectrometric (LOD = 0.0005 mg/mL) [17] assays based on stopped flow injection techniques for the detection of chlorate in soils.

### 3.2. Selectivity

**Figure 5:**
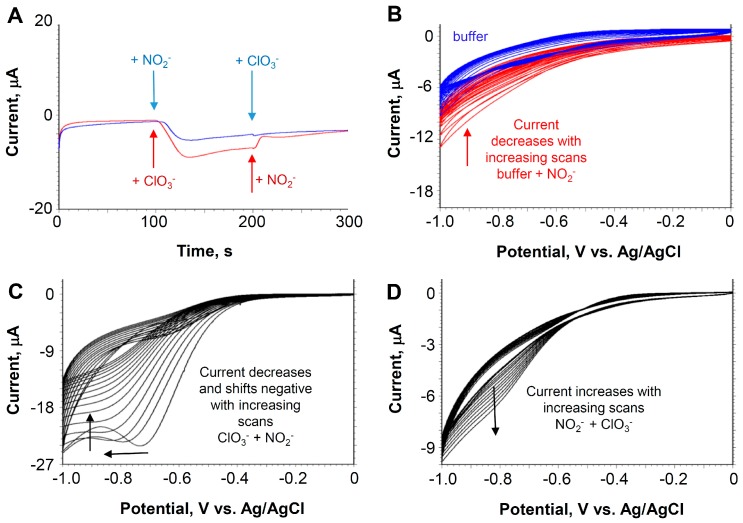
(**A**) Chronoamperometry experiments at the paper-based probe in which nitrite or chlorate (10 mM) is added in different sequences to the paper-based probe over the course of the experiment. Potential of the working electrode = −0.7 V vs. Ag/AgCl. (**B**) Cyclic voltammograms of the paper-based probe with the addition of 10 mM sodium nitrite. (**C**) Addition of 10 mM sodium nitrite after the addition and CV equilibration of 10 mM chlorate. (**D**) Addition of 10 mM potassium chlorate after the addition and CV equilibration of 10 mM sodium nitrite. For (B–D), scan rate = 100 mV/s.

Experiments were performed to see if the paper-based probe would be selective between chlorate and nitrite, since POMs are also catalytic for the reduction of nitrite [26]. Chronoamperometry experiments were performed in which nitrite or chlorate was added in different sequences to the paper-based probe over the course of the experiment. In these experiments, the working electrode was held at a fix potential of −0.7 V vs. Ag/AgCl and the current was recorded as a function of time. As shown in the blue trace in Figure 5A, at 100 s the addition of nitrite yields a reduction signal that is about half as strong as the signal for chlorate (red trace). At 200 s, the addition of chlorate after the addition of nitrite does not increase the signal significantly (blue trace), and the addition of nitrite after the addition of chlorate decreases the signal (red trace). These results suggest that nitrite will block the catalytic reduction for chlorate.

The same effect can also be shown in CV experiments. As shown in Figure 5B, with the addition of nitrite, there was an increase in current from a featureless CV for the initial signal of the reduction current, which decays to buffer levels. As shown in Figure 5C, after the chlorate signal has reached a steady state, showing a typical catalytic peak current, the addition of nitrite decreases the current and the peak shifts negative into a broad featureless catalytic trace similar to the CV present in the nitrite-only result. As shown in Figure 5D, adding chlorate after the addition of nitrite only slightly increases the catalytic reduction current. In both chronoamperometry and voltammetry experiments, it is clear that nitrite blocks the catalytic reduction of chlorate in the paper-based probe. On the other hand, nitrite and chlorate yield clear and distinguishable catalytic CV signatures, which when measured separately can be used for identification.

### 3.3. Soil Assay

**Figure 6:**
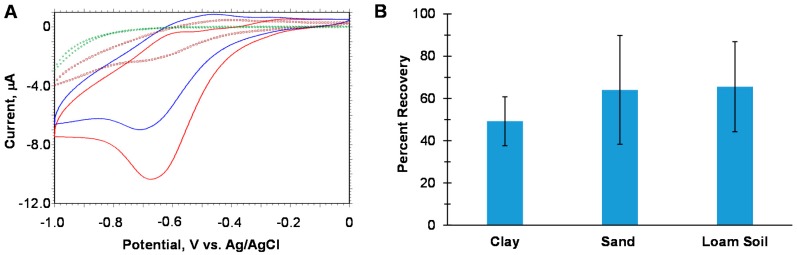
(**A**) Example cyclic voltammograms of different chlorate samples recorded at 100 mV/s for the paper-based electrochemical probe containing 0.45 mg [PMo_11_VO_40_]^5−^. The red CV is the chlorate response without sand and the blue CV is with sand. The trace with the brown squares is the CV without chlorate and the trace with the green circles is without catalyst. Conditions = 100 mg soil with 250 μg of KClO_3_ and a total volume of buffer in the assay = 180 μL, total concentration of [KClO_3_] = 1.4 mg/mL. Scans 29 and 30 are displayed for each CV. (**B**) Percent recovery of the sensor response of different soil samples.

We developed a simple CV assay for soils in which the paper can also serve as a filter for sampling, highlighting our theme of practical field work. In our assay, several soil types were spiked with KClO_3_ and directly added on top of the filter paper with buffer for a direct measurement with no sample preparation. The response from the soil was compared to the same conditions and concentrations with a buffer only sample (no soil), and a percent recovery was calculated from the ratio. Example voltammograms are shown in Figure 6A. Three soil types were tested and the assays were repeated in triplicate, with the results shown in Figure 6B. The percent recoveries are low for all soil types suggesting that the simple assay does not directly measure all the chlorate in spiked soil. This suggests that chlorate could be adsorbed onto the soil, so the buffer would have to “extract” it first to solubilize it. In addition, the chlorate would have to diffuse through a tortuous path to the electrode to be detected. The simple assay is not quantitative, but should be adequate for qualitative identification for bulk analysis in which soil has been contaminated at low percentages of chlorate at the sub-mg level in which optical methods would be challenged.

### 3.4. Digital Simulation

Interestingly, in the CV studies, an additional peak centered between −0.5 and −0.6 V begins to grow at high chlorate concentration in the anodic sweep of the CVs (Figure 1B). The direction of the current is in a negative direction, indicating a reduction. This result was not seen with any of the other salts and was only seen at high concentrations of chlorate. One possible mechanism for this phenomenon is the inactivation of the electrocatalyst at more negative reduction potential, after which, in the reverse sweep, the catalyst is regenerated, producing a cathodic peak in the reverse scan.

An attempt to simulate this phenomenon with a simplified model that is strictly qualitative was made. The simplification was necessary to keep the simulation tractable. In our model, A, which represents the polyoxometalate anion, becomes reduced to B at a mild reduction potential. The rate is slow, shifting the peak to more negative values. The active form of the electrocatalyst, B, then reduces the chlorate anion and is regenerated back to A, completing the catalytic step.

**Figure 7:**
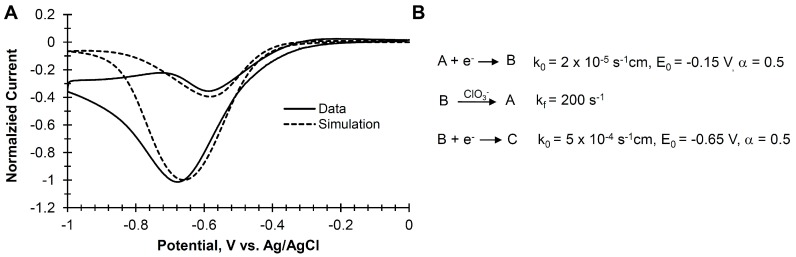
(**A**) Baseline subtracted cyclic voltammogram (solid line) at [KClO_3_] = 5 mg/mL compared to the simulated model (dotted line). (**B**) The electrochemical model used in the digital simulation.

To generate the observed phenomenon, a competing reaction was added to the model in which a second reduction at a more negative potential generates C, which is an inactive form of the electrocatalyst. In the reverse sweep, B is then regenerated from C and catalysis proceeds, generating the additional catalytic cathodic peak. The model is simulated in Figure 7 and overlaid with a background subtracted data set at [KClO_3_] = 5 mg/mL. While this model is simplistic and does not include multiple electron and atom transfer steps, it does generally reproduce the observed phenomenon and since the second cathodic catalytic peak in the return sweep is not seen with other salts, the phenomenon could be used as a descriptive signature for chlorate identification. We choose not to speculate about the nature of C here, but are pursuing further studies related to its identification and characterization for future publications.

## 4. Conclusions

The use of a paper-based probe impregnated with the vanadium-containing polyoxometalate anion, [PMo_11_VO_40_]^5−^, on top of screen-printed carbon electrodes via the electrochemical method described herein represents a simple technique for the detection of chlorate in solution. This method can be utilized in numerous electrochemical systems. For [PMo_11_VO_40_]^5−^-impregnated filter paper spiked with chlorate, the LOD for the CV assay was 0.083 mg/mL (S/N > 3). This performance was reproducible for [PMo_11_VO_40_]^5−^-impregnated paper stored under ambient conditions for at least 8 months prior to use. A simple soil assay in which different soil types spiked with KClO_3_ were directly added to the probe without any pre-sample preparation was also tested. While this simple soil assay precluded quantitative analysis, a qualitative “yes or no” answer obtained with bulk powder on soil surfaces is adequate for UAV-based chlorate identification applications. In addition, the observed phenomenon of the second cathodic catalytic peak in the return sweep, which could be used as a descriptive signature for chlorate identification, highlights the simplicity of the electrochemical assay for field work.

## Figures and Tables

**Table 1:** Analytical parameters for the detection of chlorate using a paper-based electrochemical probe containing [PMo_11_VO_40_]^5−^.

Electrochemical Technique	Linear Range (mg/mL)	Sensitivity	LOD ^a^ (mg/mL)
Cyclic voltammetry, I_pc_ vs. dose	0.156–1.25	8.2 μA per 1 mg/mL	0.083
Chronocoulometry, charge vs. dose	0.625–2.5	2.6 mC per 1 mg/mL	0.31
Chronocoulometry, slope (charge/s) vs. dose	0.312–2.5	0.25 mC/s per 1 mg/mL	0.15

^a^ The limit of detection (LOD) was calculated using the equation LOD = 3 s/m, where s = the standard deviation of the lowest concentration measured and m = the slope of the linear region for the dose–response curve [25].
